# Silver nanoparticles as next-generation antimicrobial agents: mechanisms, challenges, and innovations against multidrug-resistant bacteria

**DOI:** 10.3389/fcimb.2025.1599113

**Published:** 2025-08-14

**Authors:** Hazim O. Khalifa, Atef Oreiby, Temesgen Mohammed, Mohamed A. A. Abdelhamid, Essam Nageh Sholkamy, Hamada Hashem, Ragab M. Fereig

**Affiliations:** ^1^ Department of Veterinary Medicine, College of Agriculture and Veterinary Medicine, United Arab Emirates University, Al Ain, United Arab Emirates; ^2^ United Arab Emirates University (UAEU) Center for Public Policy and Leadership, United Arab Emirates University, Al Ain, United Arab Emirates; ^3^ Department of Animal Medicine, Faculty of Veterinary Medicine, Kafrelsheikh University, Kafrelsheikh, Egypt; ^4^ Biology Department, Faculty of Education and Arts, Sohar University, Sohar, Oman; ^5^ Department of Biotechnology and Bioinformatics, Korea University, Sejong, Republic of Korea; ^6^ Department of Botany and Microbiology, College of Science, King Saud University, Riyadh, Saudi Arabia; ^7^ Department of Pharmaceutical Chemistry, Faculty of Pharmacy, Sohag University, Sohag, Egypt; ^8^ Division of Internal Medicine, Department of Animal Medicine, Faculty of Veterinary Medicine, South Valley University, Qena, Egypt

**Keywords:** silver nanoparticles, antimicrobial mechanisms, antibiotic resistance, multidrug resistance, targeted delivery, nanomedicine, controlled release

## Abstract

The escalating prevalence of multidrug-resistant (MDR) bacteria presents a critical global health challenge, necessitating the urgent development of alternative antimicrobial strategies. Silver nanoparticles (AgNPs) have emerged as promising antimicrobial agents due to their broad-spectrum activity, unique physicochemical properties, and multiple mechanisms of bacterial inhibition. Their nanoscale size, high surface area-to-volume ratio, and ability to generate reactive oxygen species (ROS) make them highly effective against both Gram-positive and Gram-negative bacteria. AgNPs exert their antimicrobial effects through diverse mechanisms, including membrane disruption, protein and DNA interactions, enzymatic inhibition, and interference with bacterial metabolic pathways. Despite their potent antibacterial activity, concerns regarding bacterial adaptation, cytotoxicity, and non-specific interactions have prompted extensive research into innovative delivery systems to enhance AgNP efficacy while minimizing adverse effects. This review comprehensively explores the synthesis methods and physical properties of AgNPs, emphasizing their antimicrobial mechanisms and emerging resistance patterns. Additionally, we discuss advanced targeted delivery approaches, including surface functionalization, biopolymer encapsulation, liposomal carriers, and stimuli-responsive nanoplatforms, which enhance the stability, selectivity, and controlled release of AgNPs. These strategies not only improve AgNP bioavailability but also reduce host toxicity and prevent bacterial resistance development. Furthermore, we highlight future directions in AgNP-based antimicrobial therapy, such as combinatorial treatments with antibiotics, advanced nanostructure modifications, and the integration of AgNPs into wound dressings, coatings, and biomedical devices. By synthesizing recent advancements, this review underscores the transformative potential of AgNPs as next-generation antimicrobial agents to combat MDR bacterial infections. Addressing the current limitations and optimizing AgNP formulations will be crucial for their successful clinical translation and for mitigating the global antibiotic resistance crisis.

## 1 Introduction

Antibiotics, which are substances effective against bacteria, were first discovered by Alexander Fleming in 1928 (Fleming, 1929). These drugs have since become indispensable in modern medicine, as well as in various other sectors, including agriculture and the food industry (Capita and Alonso-Calleja, 2013). Antimicrobial resistance (AMR) refers to the process by which microorganisms—such as bacteria, viruses, fungi, and parasites—develop resistance to the drugs designed to combat them. The primary driver of AMR is the excessive and improper use of antibiotics in humans, animals, and the environment (Ahmed et al., 2015; Khalifa et al., 2022a). This growing problem is largely attributed to the overuse of antibiotics in human and veterinary fields, which has become a critical global public health concern (Khalifa et al., 2021a; Oreiby et al., 2019). AMR makes infections harder to treat, increasing the risk of disease transmission, severe illness, and death (Khalifa et al., 2025; Khalifa et al., 2024b). According to a recent comprehensive study, bacterial AMR was linked to approximately 4.95 million deaths in 2019, with an estimated 1.27 million of those deaths directly caused by bacterial AMR (95% uncertainty interval: 0.911–1.71 million) (Murray et al., 2022). Therefore, several international organizations, including the Food and Agriculture Organization (FAO), have begun publishing country-specific guidelines for antimicrobial use and raising awareness about this issue (Hegazy and Oreiby, 2024).

The demand for unconventional antibiotics has become an essential focus for modern antibiotic researchers, who are working to navigate the evolving challenges of bacterial pathogenesis, particularly in relation to Gram-negative bacteria (Khalifa et al., 2022b; Al-Hakkani et al., 2023). This approach aims to tackle problems that initially seem insurmountable. Encouragingly, a recent review of the global preclinical antibacterial pipeline reveals a significant surge in activity related to unconventional treatments (Theuretzbacher et al., 2019). Although nontraditional therapies show promise for the future, demonstrating their clinical effectiveness will require substantial funding. This is especially true given that many of the translational demands for small, direct-acting molecules are unlikely to align with the alternative development pathways. Moreover, unconventional treatments are more likely to be used alongside antibiotics than as stand-alone therapies. This can complicate clinical trials by making it difficult to attribute outcomes to the new agent, potentially affecting efficacy assessments and regulatory approval (Theuretzbacher and Piddock, 2019).

Currently, there is widespread evidence that nanoparticles could serve as a promising alternative to antibiotics, potentially offering significant help in tackling the issue of bacterial multi-drug resistance (Rai et al., 2012). In particular, AgNPs have attracted considerable attention within the scientific community (Baveloni et al., 2025; Aljowaie and Aziz, 2025; Al-Asbahi et al., 2024). In recent years, AgNPs have been viewed as particularly promising for the development of a new class of antibiotics, offering a novel approach to combat a variety of bacterial infections (Caniglia et al., 2024; Ibraheem et al., 2024; Iwuji et al., 2024; Tunç, 2024; Soltani et al., 2024; Yiğit et al., 2024). This review aims to provide an in-depth analysis of the potential of AgNPs as antimicrobial agents against multidrug-resistant (MDR) bacteria. It will explore the synthesis methods, physical properties, and antimicrobial activities of AgNPs. Furthermore, we will discuss the mechanisms by which AgNPs exert their bactericidal effects and address emerging concerns related to bacterial resistance to AgNPs. We will also review advanced strategies for targeted delivery, which aim to improve the selectivity and effectiveness of AgNPs while reducing cytotoxicity. Finally, we will highlight future directions for enhancing AgNP-based antimicrobial therapies, focusing on innovative methods to optimize their clinical use. Through this detailed review, we hope to offer valuable insights into the potential of AgNPs as a novel and effective solution for treating MDR bacterial infections.

## 2 Synthesis of AgNPs

The synthesis of AgNPs can be achieved through various approaches, including physical, chemical, and green synthesis methods.

### 2.1 Physical and chemical synthesis

Physical methods, such as evaporation-condensation, spark discharge, and pyrolysis, offer rapid synthesis without hazardous chemicals but suffer from high energy consumption, low yield, and inconsistent particle distribution (Kruis et al., 2000; Zhang et al., 2016; Elsupikhe et al., 2015; Xu et al., 2020).

**Figure 1:**
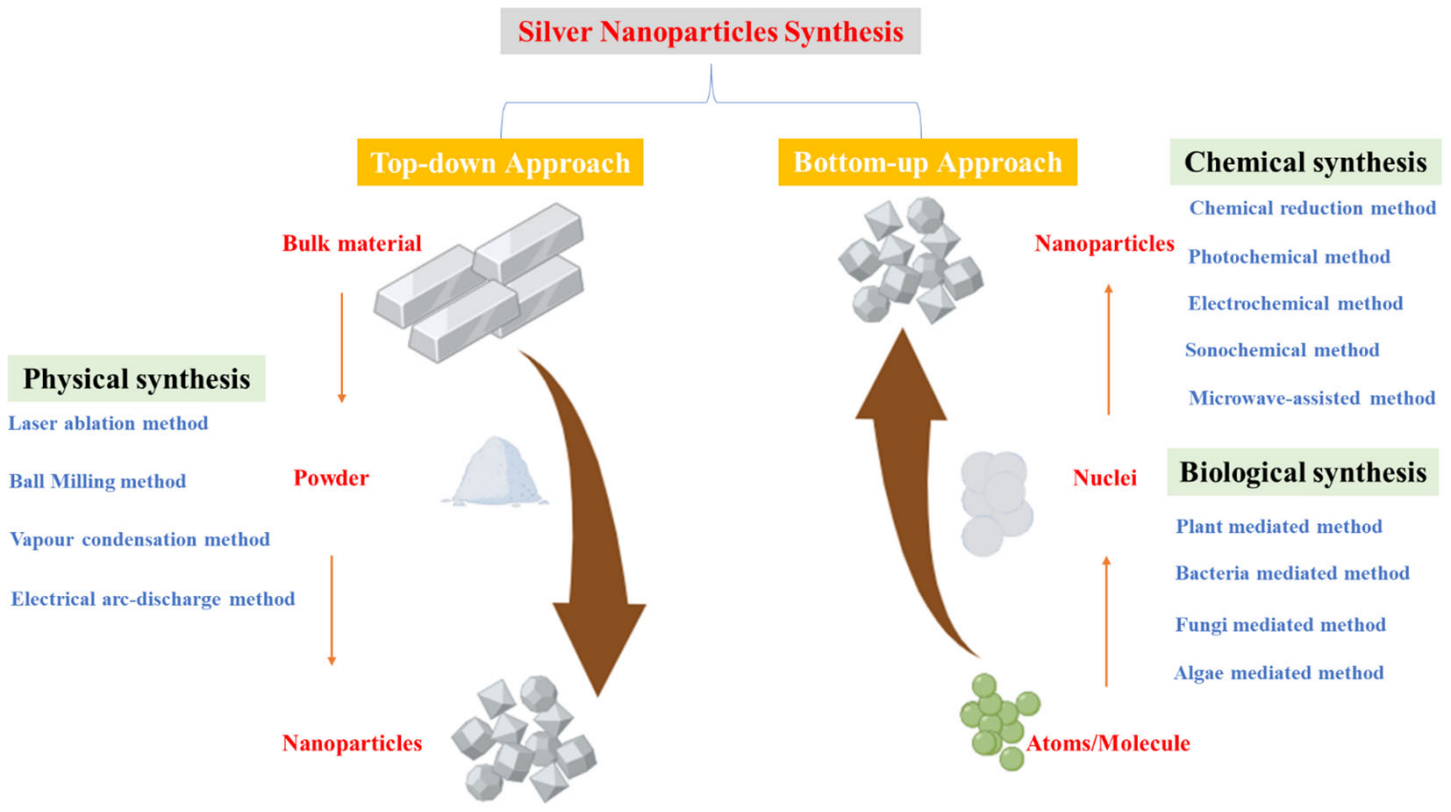
Synthesis of silver nanoparticles can be categorized into two main approaches: the top-down and bottom-up methods. The top-down approach involves breaking down bulk metal materials into nanoparticles, whereas the bottom-up approach focuses on assembling nanoparticles from molecular components, leading to the formation of complex clusters. These approaches encompass various synthesis techniques, including physical, chemical, and biological methods, each employed separately. Adapted with modifications from (Xu et al., 2020).

Chemical synthesis, which involves metal precursors, reducing agents, and stabilizers, follows either a “top-down” (mechanical grinding) or “bottom-up” (chemical reduction, electrochemical methods) approach (**Figure 1**) (Amulyavichus et al., 1998; Mallick et al., 2004; Xu et al., 2020). While this method provides high yield, it is costly, involves toxic reagents (e.g., citrate, borohydride), and requires additional purification steps to prevent contamination and aggregation (Malik et al., 2002; Mallick et al., 2004). Chemical methods include laser ablation, lithography, electrochemical reduction, thermal decomposition, and sono-decomposition (Zhang et al., 2016). Despite their efficiency, chemical synthesis poses environmental and biological risks due to toxic byproducts (Gurunathan et al., 2015).

### 2.2 Green synthesis

To address the limitations of chemical methods, biological synthesis has emerged as an eco-friendly and cost-effective alternative. This method utilizes bacteria, fungi, plant extracts, and biomolecules like amino acids and vitamins to produce AgNPs in a controlled manner (Gurunathan et al., 2013, 2014; Zhang et al., 2016). Early studies on bacterial bio-sorption of metals indicated the potential for nanoparticle synthesis, though initial results were aggregates rather than discrete nanoparticles (Mullen et al., 1989). Various microorganisms, including *Pseudomonas stutzeri* AG259, *Lactobacillus* species, *Bacillus licheniformis*, and *Escherichia coli*, as well as fungi like *Fusarium oxysporum* and *Ganoderma neo-japonicum Imazeki*, have been used for AgNP production (Zhang et al., 2016). Plant-based synthesis has been demonstrated with *Allophylus cobbe, Artemisia princeps, and Typha angustifolia* (Gurunathan et al., 2014; Gurunathan, 2015; Gurunathan et al., 2015). Additionally, biopolymers, starch, enzymes, and amino acids serve as reducing agents (Leung et al., 2010; Deepak et al., 2011; Kumar et al., 2014; Zhang et al., 2016).

Biological synthesis methods provide improved control over nanoparticle size and shape due to the presence of natural reducing and capping agents—such as polyphenols, proteins, and flavonoids—in plant extracts, which guide uniform nucleation and growth while eliminating the need for toxic chemicals or synthetic stabilizers (Gurunathan et al., 2014; Restrepo and Villa, 2021). The use of natural reducing agents allows for monodisperse, stable, and water-soluble nanoparticles (Thakkar et al., 2010; Zhang et al., 2016). Particle morphology influences bioactivity, with smaller, triangular nanoparticles exhibiting enhanced effects (Morones et al., 2005). Unlike chemical methods, biological synthesis allows optimization of reaction conditions to achieve desired nanoparticle characteristics (Zhang et al., 2016).

Green synthesis of AgNPs has emerged as an environmentally friendly alternative to conventional physical and chemical methods. However, several challenges hinder its large-scale application and industrial adoption. One major limitation is the dependence on plant materials that are often geographically restricted or seasonally available. For instance, *Lithodora hispidula*, used for Pd NP synthesis, is only found in limited regions such as Cyrenaica and southern Turkey (Turunc et al., 2017). Similarly, plants like coconut, Acacia, and Andean blackberry, which have been employed in synthesizing Ag or Cu nanoparticles, are region-specific and not easily accessible worldwide (Ying et al., 2022). Seasonal constraints further complicate sourcing; materials like cotton leaves, peach blossoms, and *Trigonella trifoliata* seeds must be harvested during narrow windows, which limits continuous production (Ying et al., 2022). Moreover, some raw materials require additional processing or extraction steps, such as the case with carboxymethyl cellulose and tea polyphenols, which increases complexity and cost (Wang et al., 2017). These factors challenge the feasibility, cost-effectiveness, and scalability of green synthesis in industrial contexts.

In addition to raw material constraints, the synthesis process itself presents technical and environmental limitations. Many green synthesis protocols require excessive energy input, long reaction durations, or strict environmental controls, such as high-temperature treatments or inert atmospheres. For example, AgNPs have been synthesized using *Ferula persica* root extract at 600°C for 3 hours (Nasiri et al., 2018), and *Cystoseira baccata* extract was stored at −24°C to retain its reactivity (González-Ballesteros et al., 2017). These conditions contradict the principle of sustainability and elevate production costs. Furthermore, the resulting nanoparticles often exhibit high variability in size, morphology, and crystallinity. AgNPs synthesized using *Nigella arvensis* leaf extract, for example, ranged from 5 to 100 nm, while NZVI particles from grape seeds varied between 63 and 381 nm (Gao et al., 2016; Chahardoli et al., 2018). This inconsistency limits reproducibility and standardization required for commercial and biomedical applications. In many cases, yields and metal ion conversion rates remain low—often below 50%—which diminishes economic viability (Ying et al., 2022). The limited understanding of the underlying biosynthetic mechanisms further complicates optimization, as many studies only infer the roles of plant extracts (e.g., reducing, capping, or chelating agents) without detailing specific chemical pathways (Ying et al., 2022). Collectively, these limitations highlight the need for more systematic research to improve the reliability, efficiency, and industrial compatibility of green synthesis methods.

## 3 Physical properties of AgNPs in relation to antimicrobial effect

AgNPs possess unique physicochemical properties that make them highly effective as antimicrobial agents against MDR bacteria. Their physical characteristics—including size, shape, surface charge, electrical conductivity, melting point, thermal conductivity, and optical properties—strongly influence their biological interactions and antimicrobial efficacy.

### 3.1 Size and surface area

The size of AgNPs is a critical determinant of their antimicrobial activity, as it directly affects their surface-area-to-volume ratio and interaction with bacterial cells. Smaller nanoparticles exhibit enhanced bioactivity due to their increased surface area, facilitating greater silver ion release and direct interaction with microbial membranes (Khan et al., 2021). Studies have shown that AgNPs attach to bacterial cell membranes, disrupting the lipid bilayer and increasing permeability, leading to cell death—an effect more pronounced with smaller nanoparticles (Li et al., 2013). The size of AgNPs also influences their toxicity; for instance, nanoparticles below 10 nm have been shown to induce more significant cytotoxic effects in mammalian cells than larger particles. For example, AgNPs exhibit strong potency, possess small average diameters of approximately 10 nm, and demonstrate cytotoxicity in human lung cells (Gliga et al., 2014). Furthermore, Fernández et al., 2019 examined the cytotoxic effects of 10 nm and 60 nm silver nanoparticles (AgNPs) in hepatic (HepG2) cells, a model chosen due to the liver’s known tendency to accumulate AgNPs. Their findings showed that 10 nm AgNPs could enter the nucleus, whereas 60 nm particles remained aggregated in the cytoplasm. Proteomic analysis revealed that nearly 50 proteins were altered by each nanoparticle size, but only four showed similar regulation patterns, suggesting that distinct cellular pathways were triggered depending on particle size (Fernández et al., 2019). Complementing this, Wu et al., 2019 studied AgNPs of various diameters (3.2, 20.7, 54.7, and 93.6 nm) in B16 mouse melanoma cells and found that nanoparticle size not only affected uptake efficiency but also dictated the endocytic pathways involved. These findings highlight the importance of nanoparticle size in determining both cellular entry routes and biological responses.

### 3.2 Shape and antimicrobial efficacy

The morphology of AgNPs significantly affects their interaction with bacterial membranes and their overall antimicrobial properties. AgNPs can be synthesized in various shapes, including spherical, triangular, cubic, rod-shaped, and star-like structures (Khodashenas and Ghorbani, 2019). Studies have demonstrated that the shape of silver nanoparticles (AgNPs) plays a critical role in determining their antimicrobial efficacy. For instance, Alshareef et al. (2017) reported strong antibacterial activity of both rod- and spherical-shaped AgNPs. Similarly, Cheon et al. (2019) synthesized AgNPs in three shapes—spherical, disc-shaped, and triangular—and observed the highest bactericidal activity in spherical forms, followed by disc-shaped and then triangular nanoparticles. In contrast, other studies have highlighted the superior antibacterial properties of triangular nanoparticles due to their sharp edges, which enhance interactions with bacterial membranes and lead to mechanical disruption (Liao et al., 2019). These variations suggest that the antibacterial effectiveness of AgNPs may depend on multiple factors, including bacterial type, environment, and application. Additionally, comparative studies have shown that rod-shaped AgNPs penetrate bacterial biofilms more effectively than spherical particles, making them particularly useful for combating biofilm-associated MDR infections (Wang et al., 2021). Other studies have compared the antimicrobial activities of spherical, rod-shaped, and truncated triangular silver nanoplates against planktonic *E. coli* cells, revealing that truncated triangular AgNPs exhibit the strongest bactericidal effect. This enhanced activity is attributed to the greater number of facets on triangular nanoparticles, which allows for increased interaction with bacterial surfaces, leading to more extensive membrane damage (Pal et al., 2007). This difference is likely due to the higher aspect ratio of rod-shaped nanoparticles, which enhances their interaction with biofilms (Slomberg et al., 2013). This variation in antimicrobial activity is likely due to differences in silver ion release, which is influenced by the surface area of the nanoparticles. Consequently, they concluded that modifying the morphology of AgNPs can effectively regulate their antimicrobial efficacy.

### 3.3 Surface charge and stability

Surface charge plays a crucial role in the stability and biological interactions of AgNPs. The zeta potential of AgNPs determines their colloidal stability, with highly positive or negative values preventing aggregation (Bélteky et al., 2019). Positively charged AgNPs exhibit stronger interactions with bacterial cell membranes, which are typically negatively charged due to the presence of lipopolysaccharides and teichoic acids (Franco et al., 2022). This electrostatic attraction enhances bacterial membrane penetration and increases antimicrobial activity. For example, Abbaszadegan et al. investigated the impact of AgNP surface charge on antimicrobial activity against Gram-positive (*S. aureus*, *S. mutans*, and *S. pyogenes*) and Gram-negative (*E. coli* and *P. vulgaris*) bacteria (Abbaszadegan et al., 2015). Their findings revealed that positively charged AgNPs exhibited the strongest bactericidal effect against all tested strains, while negatively charged AgNPs demonstrated the weakest activity. Neutral AgNPs displayed an intermediate level of antibacterial effectiveness. Functionalization of AgNPs with biocompatible polymers such as chitosan or polyethyleneimine further improves stability and bioavailability while reducing cytotoxicity to human cells (Liao et al., 2019).

### 3.4 Electrical conductivity and melting point

To date, numerous nanomaterials have been developed with the potential to serve as transducing elements, owing to their superior electrical conductivity, as well as enhanced thermal and optical properties (Ventura-Aguilar et al., 2023). AgNPs exhibit a reduced melting point compared to bulk silver due to their high surface energy and increased atom mobility (Asoro et al., 2009). This property is particularly beneficial in antimicrobial coatings for medical devices, as it enables the formation of conductive and biocidal nanocomposites at lower processing temperatures (Abbas et al., 2024). The high electrical conductivity of AgNPs also facilitates their integration into biosensors for rapid detection of bacterial infections (Ventura-Aguilar et al., 2023).

### 3.5 Thermal conductivity

AgNPs exhibit remarkable thermal conductivity, making them highly suitable for heat-based antimicrobial applications. In previous studies, the researchers explored their potential as multifunctional agents to enhance hyperthermia, directly eliminate cancer and bacterial cells, or function as photothermal therapy agents (Liu S. et al., 2023). The findings demonstrated the effectiveness of AgNPs in eradicating both breast cancer cells and bacteria within the breast tumor microenvironment. Recent studies also have highlighted the role of AgNP-based nanocomposites in improving heat transfer efficiency in antimicrobial surface coatings, and reducing bacterial colonization on biomedical implants (Sahoo et al., 2022).

### 3.6 Optical properties and plasmon resonance

AgNPs exhibit unique optical properties due to localized surface plasmon resonance (LSPR), where conduction electrons oscillate in response to incident light (Loiseau et al., 2019). This property makes AgNPs highly effective in biosensing and antimicrobial photodynamic therapy (Gabudean et al., 2011). This phenomenon enhances the generation of ROS, which contribute to bacterial cell membrane disruption and oxidative stress-induced cell death (Canaparo et al., 2020). Additionally, LSPR facilitates photothermal and photodynamic effects, where AgNPs absorb light energy and convert it into localized heat, further aiding in bacterial eradication (Liu H. et al., 2023). The plasmonic properties of AgNPs can also be tuned by modifying their size, shape, and surrounding environment, allowing for optimized antimicrobial effects (Vasil’kov et al., 2018). These optical characteristics make AgNPs promising candidates for developing light-activated antimicrobial therapies against multidrug-resistant bacteria.

## 4 Antimicrobial activities of AgNPs

AgNPs have demonstrated potent antimicrobial effects against a wide range of pathogenic microorganisms, including both Gram-positive and Gram-negative bacteria, as well as fungi (**Table 1**). Their effectiveness varies based on their synthesis method, particle size, and microbial target. Most studies report spherical nanoparticles with sizes typically between 5–100 nm. While antimicrobial effects were consistently observed across different concentrations and testing methods, detailed investigations into their mechanisms of action remain limited (**Table 1**).

**Table 1:** Characteristics, antimicrobial activities, and mechanism of action of silver nanoparticles.

Nanoparticles	Description	Shape	Size (nm)	Tested concentrations	Species/Strain	Method to evaluate antimicrobial effect	Mechanism of action	Method to evaluate the mechanism of action	References
AgNPs	AgNPs were produced via a chemical synthesis method.	Spherical	24.3 ± 0.18	0.02 μg/mL to 58.5 μg/mL	*S. aureus*, *P. aeruginosa*, and *E. coli*	Broth microdilution assay	Not determined	Not determined	Baveloni et al. (2025)
AgNPs from Teucrium polium leaves	Silver nanoparticles were green-synthesized using *Teucrium polium* leaf extract.	Spherical	41 to 61	500, 1,000, and 1,500 μg/mL	*S. aureus* (MTCC-29213), *B. subtilis* (MTCC-10400), *S. epidermidis* (MTCC-12228), *E. coli* (ATCC-25922), *K. pneumoniae* (MTCC-13883), and *P. aeruginosa* (MTCC-27853)	Disk diffusion and broth dilution assays	Not determined	Not determined	Aljowaie and Aziz (2025)
B-AgNPs, L-AgNPs, and LB-AgNPs	AgNPs were biosynthesized using a combination of *Lactobacillus* sp. and *Bacillus* sp. growth.	Spherical	B-AgNPs: 11–22.8, L-AgNPs: 7.97–14.3, and LB-AgNPs: 4.65–11.3	10, 20, and 40 μg/mL	*P. aeruginosa* and *S. aureus*	Disk diffusion assay	Not determined	Not determined	Al-Asbahi et al. (2024)
AgNPs-PDA	AgNPs were incorporated into biocompatible catecholamine-based polymers (PDA) through localized electrochemical deposition using a double potentiostatic method via scanning electrochemical cell microscopy (SECCM).	Multiple shapes (truncated tetrahedral, dendritic, octahedral)	Average particle size of 171 ± 4	0.05 mmol L^−1^	*E. coli*	Not determined	The bacterial outer membrane exhibited structural changes, including increased hydrophilicity and reduced stiffness, when in close proximity to the AgNPs.	Atomic force microscopy (AFM)-based force spectroscopy	Caniglia et al. (2024)
AgNPs	AgNPs were produced via a chemical synthesis method.	Spherical	58.3	0.25 to 2.0 μg/mL	*S. aureus*, *P. aeruginosa*, and *E. coli*	Broth microdilution assay	Not determined	Not determined	Iwuji et al. (2024)
AgNPs from Teucrium Parvifolium	Green nanoparticles were synthesized from the aqueous extract of *Teucrium Parvifolium* plant seeds.	Spherical and crystalline	14	2, 4, 8, 16, 32, 64, 128 mg/ml for disk diffusion assay and 128 mg/ml for broth dilution assay	*E. coli* O157:H7 (ATCC No. 25922), *E. faecali*s (ATCC No. 19433), *P. aeruginosa* (ATCC No. 27853), *S. aureus* (ATCC No. 25923).	Disk diffusion and broth dilution assays	Not determined	Not determined	Soltani et al. (2024)
AgNPs	AgNPs were phytosynthesized using *Aloe vera* extract.	Spherical and scattered	42.553 ± 12.855	Up to 256 µg/mL	*E. coli*, *P. aeruginosa*, *A. baumannii*, and *S. aureus*	Broth dilution assay	Not determined	Not determined	Yiğit et al. (2024)
AgNPs-PEG-NYS	AgNPs were conjugated with PEG and Nystatin (AgNPs-PEG-NYS) using a chemical precipitation method.	Spherical	37.658, 52.328, and 71.525 for AgNPs, AgNPs-NYS, and AgNPs-PEG-NYS, respectively.	50 µg/mL	*S. aureus* and *E. coli*	Agar well diffusion assay	Not determined	Not determined	Ibraheem et al. (2024)
AgNPs and carboplatin-loaded silver nanoparticles (AgNPs-Car)	AgNPs and AgNPs-Car were synthesized via a chemical method.	Not determined	AgNPs: 6.5 and AgNPs-Car: 28.85 nm– 43.82	Up to 100 µg/mL	*E. coli* (ATCC 25922), *K. pneumoniae* (ATCC 13883), *A. baumanii* (ATCC 17978), *P. aeruginosa* (ATCC 27853), *S. aureus* (ATCC 29213), Methicillin-resistant *S. aureus* (ATCC 43300), *E. faecalis* (ATCC 29212), *B. cereus* (ATCC 11778), *C. albicans* (ATCC 10231), and *C. tropicalis* (ATCC 4563)	Broth microdilution assay	Not determined	Not determined	Tunç (2024)
AgNPs from Lepidium draba L. leaves	Silver nanoparticles were green-synthesized using *Lepidium draba L*. leaves.	Spherical	20–35	62.5–1000 μg/mL	*E. coli*, *K. pneumoniae*, *S. aureus*, *E. faecalis*, *C. albicans*	Broth microdilution assay	Not determined	Not determined	Hajizadeh et al. (2024)
Polymer film/AgNPs	A natural polymer film with AgNPs biosynthesized using aqueous plant root extracts of *Symphyti radix*.	Spherical	27.45	Not determined	*S. aureus* (ATCC 25923), Beta-hemolytic *streptococcus* group b (β-*streptococcus*) ATCC 15185, *S. epidermidis* ATCC 12228, *E. faecalis* ATCC 29212, *E. coli* ATCC 25922, *K. pneumoniae* ATCC 13883, *P. aeruginosa* ATCC 27853, *P. vulgaris* ATCC 8427, *B. cereus* ATCC 11778, and *C. albicans* ATCC 10231	Agar well diffusion assay	Not determined	Not determined	Balciunaitiene et al. (2024)
AgNPs_mPEG_AK	AgNPs functionalized with mercaptopoly(ethylene glycol) carboxylic acid (mPEG-COOH) and amikacin (AK)	Spherical	17.02 ± 1.25	AgNPs_mPEG at a final concentration of 12.5 mg/L Ag^+^; AgNPs_mPEG_AK containing 0.5 mg/L AK and 12.5 mg/L Ag^+^; a combination of AgNPs_mPEG (12.5 mg/L Ag^+^) with AK; and AK alone were each subjected to serial two-fold dilutions	12 clinical multidrug-resistant /extensively drug-resistant isolates of *A. baumannii*, *E. coli*, *K. pneumoniae*, and *P. aeruginosa*	Microdilution assay	Not determined	Not determined	Palau et al., 2023
OLAgNPs	AgNPs were green-biogenically synthesized using polyphenolic extract of olive leaf wastes.	Spherical	20–45	5, 25, 50, and 100 µg/mL	*L. monocytogenes*, *B. cereus*, *S. aureus*, *E. coli*, *Y. enterocolitica*, and C. jejuni	Disk diffusion assay	Not determined	Not determined	Alowaiesh et al. (2023)
AgNPs	Different commercially available AgNPs	Not determined	2.53 ± 1.71 and 3.06 ± 2.04	Not determined	*E. coli, P. aeruginosa, S. aureus, A. baumannii, Salmonella* spp.*, K. pneumoniae, and C. freundii*	Microdilution assay	Ag may bind bacterial fimbriae and affect cell permeability	Transmission electron microscopy	Dove et al., 2023
AN-AgNPs	AgNPs were green-synthesized using *Argyreia nervosa* leaf extract.	Not determined	10–40	Not determined	Enteropathogenic *E. coli*	Disk diffusion assay	Not determined	Not determined	Parvathalu et al. (2023)
AgNP-His	AgNPs were biosynthesized using *Lippia abyssinica* plant leaf extract.	Spherical	5–14	62.5 μg/mL	*S. aureus* ATCC 25926 and *E. coli* ATCC 25922	Agar well diffusion assay	Not determined	Not determined	Shumi et al. (2023)
AgNPs	Silver nanoparticles were biosynthesized using marine fungi: *Penicillium simplicissimum*, *Aspergillus terreus*, *Aspergillus japonicus*, and *Aspergillus oryzae.*	Spherical	3.8–23	2, 5, and 8 mM	*E. coli*, *K. pneumoniae*, *P. vulgaris*, *S.* Typhi, *E. faecalis*, *S. aureus* methicillin-resistant, *S. hominis*, and *S. epidermidis*	Agar well diffusion assay	Not determined	Not determined	Basheer et al. (2023)
AgNPs from *Citrus limon* (L.)	Biogenic synthesis using aqueous zest extract (*Citrus limon*)	Spherical and cubic	7–28	1 mg/mL	*S. aureus, E. coli, C. albicans*	Agar well diffusion assay	Not determined	Not determined	Khane et al. (2022)
AgNPs from *Gardenia thailandica*	Green synthesis utilizing leaf extract (*Gardenia thailandica*)	Spherical	11.02–17.92	Up to 250 µg/mL for microdilution assay	*S. aureus*	Microdilution and time-killing assays and *in vivo* study in rats	Bacterial cell membrane disruption, leading to shape alteration	Membrane permeability assay, SEM	Attallah et al. (2022)
ML-AgNPs (*Morinda lucida*)	Biosynthesis using leaf extract (*Morinda lucida*)	Spherical and rough-edged crystallite	11	Not determined	*Citrobacter*, *E. coli*, *P. vulgaris*, *S.* Typhi, *V. cholerae*, *E. faecalis*	Disk diffusion	Not determined	Not determined	Labulo et al. (2022)
AgNPs from *Myrsine africana*	Green synthesis from leaf extract (*Myrsine Africana*)	Spherical and oval	28.32	0.03, 0.05, 0.09, 0.11, and 0.13 mg/mL	*P. aeruginosa, S. aureus, E. coli, K. pneumoniae*,	Agar well diffusion	Not determined	Not determined	Sarwer et al. (2022)
AgNPs from *Syzygium cumini*	Biosynthesis from fruit extracts (*Syzygium cumini*)	Nearly spherical	47	25, 50, and 75 µg/mL	*S. aureus, B. subtilis, P. aeruginosa, E. coli*	Disk diffusion	Not determined	Not determined	Chakravarty et al. (2022)
AgNPs from *Hypericum perforatum L.*	Green synthesis utilizing aqueous extract (*Hypericum perforatum L)*	Spherical, monodisperse, face-centered cubic (fcc) crystal structures	20–40	50 and 100 μg/well for well diffusion assay, up 100 μg/mL for broth dilution assay, and 1, 3, 6, 12, and 24 μg/mL for growth curve assays	*P. aeruginosa*, *K. pneumoniae* (β-lactamase-producer), *E. coli* (ESBL-producers), *E. coli* (ATCC 25922), *S. aureus* (ATCC 43300), *B. cereus* (ATCC 11778), *B. subtilis* (ATCC 6633)	Well diffusion, broth dilution, growth curve assays	Not determined	Not determined	Alahmad et al. (2022)
GT AgNPs (*Green tea*)	Green synthesis using tea leaf extract (*Green tea*)	Spherical	15–33	10, 20, and 50 mg/ml	*S. aureus, Klebsiella* sp.	Disk diffusion assay	Not determined	Not determined	Widatalla et al. (2022)
AgNPs-LCg & AgNPs-FCg (*Calotropis gigantea*)	Phytosynthesis using leaf and flower extracts (*Calotropis gigantea*)	Spherical	163.5–256.7, 188.35–227.65	2, 5, or 9 mM	*E. coli, S. aureus*, and *C. albicans*	Disk diffusion assay	Not determined	Not determined	Kemala et al. (2022)
Chi/AgNPs	Chitosan-stabilized AgNPs	Nearly spherical	9–65	Up to 200 μg/mL for broth dilution assay	*S. aureus, P. aeruginosa*	Broth dilution assay	Not determined	Not determined	Shehabeldine et al. (2022)
GCL-AgNPs (*Glochidion candolleanum*)	Phytosynthesis using leaf extract (*Glochidion candolleanum*)	Spherical and ellipsoidal	Not specified	0.3, 0.5, and 1 mg/ml	*B. subtilis, L. monocytogenes, S. aureus, E. coli, P. aeruginosa, S. enterica*	Well diffusion	Not determined	Not determined	Balachandar et al. (2022)
AgNPs from *Zingiber zerumbet*	Green synthesis using wild ginger extract (*Zingiber zerumbet*)	Spherical	24.28–153.2	100 µg/ml for agar well diffusion assay and Concentrations of 50, 25, 12.5, 6.25, 1.56, 0.78.0.39, 0.195, and 0.097 µg/mL for broth microdilution assay	*S. aureus, E. faecalis, E. mutans*	Agar well diffusion and broth microdilution	Not determined	Not determined	Ramzan et al. (2022)
AgNPs from *Rubus ellipticus Sm.*	Biogenic synthesis from root extract (*Rubus ellipticus Sm*)	spherical and monodispersed	13.85 - 34.30 with an average of 25.20 ± 7.01	Not determined	*E. coli, S. aureus, K. pneumoniae, E. faecalis*	Agar well diffusion assay	Not determined	Not determined	Khanal et al. (2022)
AgNPs-BM & AgNPs-WM (*Agaricus bisporus*)	Biosynthesis using brown & white mushroom extracts (*Agaricus bisporus*)	Spherical	5 (BM), 11 nm (WM)	Not determined	*S. aureus, S. epidermidis, B. subtilis, E. coli, S.* Typhi*, P. aeruginosa*	Agar well diffusion assay	Not determined	Not determined	Al-Dbass et al. (2022)
MOAgNPs (*Moringa oleifera*)	Biogenic synthesis from aqueous leaf extract (*Moringa oleifera*)	Spherical	5–50	10 and 20 μg/mL	*E. coli, S. marcescens, S. aureus, B. subtilis*	Disk diffusion	Not determined	Not determined	Abdel-Rahman et al. (2022)
EC-AgNPs & TA-AgNPs (*Eucalyptus camaldulensis, Terminalia arjuna*)	Green synthesis using plant extracts (*Eucalyptus camaldulensis, Terminalia arjuna*)	Spherical	100 (EC), 35 (TA)	Not determined	*B. subtilis, S. aureus, E. coli, P. multocida*	Agar well diffusion assay	Not determined	Not determined	Liaqat et al. (2022)
bAgNPs (*Syzygium cymosum*)	Biogenic synthesis using plant extract (*Syzygium cymosum*)	Spherical	17.2–35.3	0.125, 0.25, 0.5, 1, 2, 3, 3.5, 5, and 6 μg/ml for broth dilution assay	*B. subtilis, E. coli* DH5α*, E. coli* K12*, enteropathogenic E. coli, S.* Typhi	Disk diffusion, broth dilution	Lipid peroxidation	Lipid peroxidation assay	Mahmud et al. (2022)
AgNPs (Various sources)	Phytosynthesis using extracts from apple, orange, potato, red pepper, onion, garlic, radish	Spherical	9–30	0.004 to 42.25 μg/ml	*S. aureus (ATCC 6538), B. cereus (ATCC 10987), E. coli (ATCC 11229)*	Broth dilution	Not determined	Not determined	Wasilewska et al. (2022)
AgNPs-KP (*Klebsiella pneumoniae*)	Green synthesis from *K. pneumoniae*	Heterogeneous with rough surface	38.9	0.009 –5000 µg/mL	*K. pneumoniae* carbapenemase-producing	Broth dilution	Not determined	Not determined	Chuy et al. (2022)
Bio-AgNPs (*Nocardiopsis dassonvillei*)	Biosynthesis using marine actinobacterium (*Nocardiopsis dassonvillei*)	Spherical	29.28	50, 100, 150, and 200 μg/ml	*S. aureus, CoNS, P. aeruginosa, ESBL-producing E. coli, Salmonella, K. pneumoniae, P. mirabilis*	Agar well diffusion assay	Not determined	Not determined	Khalil et al. (2022)
AgNPs (*Aloe vera*)	Green synthesis using *Aloe vera* extract	Hexagonals	9.26–31.18	The tested concentrations were not identified, but MIC was 85 μg/ml and MBC was 127.5 μg/ml	Multidrug-resistant *E. coli* U12	Broth dilution	Anti-biofilm activity, cell wall and membrane damage	Electron microscopy	Selem et al. (2022)
AgNPs of *Cymbopogon citratus*	Biosynthesis of AgNPs with *Cymbopogon citratus* leaf extract	Not specified	47	50, 100, 150, 200, and 250 μg/mL for broth dilution	*S.* Typhi*, B. cereus and S. flexneri*	Agar well diffusion assay and broth dilution assays	Not determined	Not determined	Rakib-Uz-Zaman et al. (2022)
AgNPs of *Talaromyces purpureogenus*	Green synthesis of AgNPs using fungus *Talaromyces purpureogenus* isolated from Taxus baccata Linn.	Spheric	30–60	25, 50, 75, and 100 μg/mL	*E. coli, S.* Typhi*, L. monocytogenes and S. dysenteriae*	Disk diffusion assay	Not determined	Not determined	Sharma et al. (2022)
AgNPs of *Anagallis monelli*	AgNPs biosynthesized using *Anagallis monelli*	Face-centered cubic structure	22	The tested concentration not mentioned, but MIC values vary from 2.812 to 11.25 mg/mL	*E. coli, K. pneumoniae, S. marcescens, S. aureus and M. luteus*	Agar well diffusion and broth dilution assays	various mechanisms such as bactericide, fungicide effects, lysozyme and anti-biofim activities as well as morphological modifications of cells	Lysozyme activity determination, Anti-biofilm activity determination, and microscopic observation to detect effect on cell viability and morphology of Candida albicans	Dridi et al. (2022)
AgNPs from *Acacia cyanophylla*	Green synthesis using aqueous plant extract (*Acacia cyanophylla*)	Spherical	88.11	0.0488–50 μg/ml	*E. coli*	Broth dilution assay	Not determined	Not determined	Jalab et al. (2021)
AgNPs from *Carthamus tinctorius* L.	Green synthesis utilizing safflower waste extract (*Carthamus tinctorius*)	Spherical	8.67 ± 4.7	0.9–250 μg/ml	*S. aureus, P. fluorescens*	Broth dilution assay	Not determined	Not determined	Rodríguez-Félix et al. (2021)
Ag-NC	Nanocomposite incorporating silver nanoparticles stabilized by polysaccharides	Spherical	15	10–160 µg/ml for broth microdilution assay	*E. coli, P. aeruginosa, S. aureus, B. subtilis* as well as fungi including *C. albicans*, *A. niger*, *A. terreus*, *A. flavus*, and *A. fumigatus*	Disk diffusion and broth microdilution assays	Not determined	Not determined	Hasanin et al. (2021)
P.yAgNPs (*Pyropia yezoensis*)	Biogenic silver nanoparticles derived from *Pyropia yezoensis*	Spherical	20–22	5, 25, 50 µg/ml for agar well dilution assay and 5–400 μg/ml for broth microdilution assay	*P. aeruginosa* and *S. aureus*	Agar well dilution and broth microdilution assays	Bacterial cell death via silver ion release, disrupting cellular signaling and integrity	Fluorescence microscopy	Ulagesan et al. (2021)
L-AgNPs	Lignin-stabilized AgNPs via green synthesis	Spherical	14.01	0.1 mg/mL, 1 mg/mL, and 10 mg/mL	*E. coli* and *C. albicans*	Disk diffusion	Not determined	Not determined	Cao et al. (2021)
AgNPs from *Cynara scolymus L.*	Phytosynthesis from artichoke waste extract	Spherical	28.78	Up to 20 μg/ml	*S. aureus, E. coli, C. albicans, B. subtilis, P. aeruginosa*	Broth dilution	Not determined	Not determined	Baran et al. (2021)
AgNPs from *Trigonella foenum-graecum*	Biogenic silver nanoparticles synthesized using fenugreek seed extract	Spherical	82.53	5 and 15 μg/ml	*E. coli, S. aureus, B. cereus*	Agar well dilution	Not determined	Not determined	Awad et al. (2021)
CSE-AgNPs & PAE-AgNPs	Phytosynthesized AgNPs from *Camellia sinensis* and *Prunus africana* extracts	Spherical, forming layers	3–98 (CSE), 4–94 (PAE)	0.1 gm of AgNPs was dissolved in 0.6 ml of sterile distilled de-ionized water, followed by two-fold serial dilution of 0.05 ml	*E. coli, K. pneumoniae*	Broth dilution assay	Not determined	Not determined	Ssekatawa et al. (2021)
AgNPs from Dsr1KO, Dsr9KD, Dsr20KD	Biosynthesis utilizing *Deinococcus radiodurans* mutants	Spherical	10–20	5−15 μg/mL for *E. coli* and *P. aeruginosa* and 30−90 μg/mL for *S. epidermidis*	*P. aeruginosa, E. coli, S. epidermidis*	Broth dilution	Not determined	Not determined	Chen et al. (2021)
AgNPs from *Nocardiopsis* spp.	Biogenic synthesis using *Nocardiopsis* strain MW279108	Spherical	2–10	214 µg/ml	*B. subtilis, B. cereus, P. aeruginosa, S.* Typhimurium*, S. aureus, A. baumannii, E. coli*	Disk diffusion	Not determined	Not determined	Abada et al. (2021)
AgNPs from *Bacillus subtilis*	Green synthesis of silver nanoparticles from *B. subtilis* metabolites	Spherical	2–26	1–64 µg/mL	*E. coli, S. aureus, V. parahemolyticus, A. baumannii*	Broth dilution	Not determined	Not determined	Yu et al. (2021)
OE-Ag	Green synthesis via *Olea europaea* leaf extract	Spherical	8	50 µL of the sample solution at 250 µg/mL concentration	*P. aeruginosa*, *K. pneumoniae*, *S. aureus, B. subtilis*	Broth dilution	Disrupts bacterial cell membranes	Live/Dead staining with confocal microscopy	Sellami et al. (2021)
AgNP-S, AgNP-F, AgNP-W	Biogenic AgNPs derived from *Carduus crispus*	Not specified	131, 33, and 70	Not determined	*E. coli, M. luteus*	Agar well dilution	Not determined	Not determined	Urnukhsaikhan et al. (2021)
Sp-AgNPs	Phytosynthesis using *Salvadora persica* root extract	Spherical and rod-like	37.5	0.19 µg/mL to 25 µg/mL	*E. coli, S. epidermidis*	Broth dilution	Bacterial membrane degradation	Nuclear staining (Syto 16)	Arshad et al. (2021)
AgNPs from *Bauhinia tomentosa Linn*	Biogenic AgNPs from *B. tomentosa*	Spherical	32	Not determined	*E. coli, S. aureus, A. flavus* and *C. albicans*	Disk diffusion	Silver binding with microbial proteins (DNA gyrase, cytochrome P450, dihydrofolate reductase)	Molecular docking	Renganathan et al. (2021)
AgNPs from *Solanum xanthocarpum*	Biogenic AgNPs from fruit extract (*Solanum xanthocarpum*)	Spherical	22.45	0.08125 to 5 mg/ml for broth dilution assay	*E. coli, Shigella* spp.*, P. aeruginosa, Aeromonas* spp.	Agar well dilution and broth dilution assays	Not determined	Not determined	Pungle et al. (2021)
AgNPs/EML, AgNPs/EMF, AgNPs/EMDS	Green synthesis utilizing *Morinda citrifolia L.* (noni)	Spherical	3–11	7.5, 5.0, or 2.5 µg	*E. coli, S. aureus*	Agar well dilution	Not determined	Not determined	Morales-Lozoya et al. (2021)
AgNPs from *Cynodon dactylon*	Biogenic synthesis using *C. dactylon* leaf extract	Spherical	15	0%, 5%, 10% and 15% per hundred parts of polymer	*P. fluorescens*	Agar diffusion test and colony count method	Not determined	Not determined	Wang et al. (2021)
AgNPs from *Carissa carandas L.*	Phytosynthesis via *C. carandas* leaf extract	Not specified	Not specified	Not determined	*S. flexneri, Citrobacter* spp., *S.* Typhimurium*, E. faecalis, Gonococos* spp.	Agar well dilution and broth microdilution assays	Not determined	Not determined	Singh et al. (2021)
Sb-AgNP	Green synthesis using *Scutellaria barbata* extract	Spherical	20–40	20, 40 and 60 µg/ml for disc diffusion	*E. coli, P. aeruginosa, S. aureus, K. pneumoniae*	XTT reduction and disc diffusion assays	Not determined	Not determined	Veeraraghavan et al. (2021)
AgNPs from *Phyllanthus emblica*	Biosynthesis using fruit extract (*Phyllanthus emblica*)	Spherical	19–45	10 μg, 20 μg, 30 μg, 40 μg and 50 μg	*K. pneumoniae, S. aureus*	Disk diffusion	Not determined	Not determined	Renuka et al. (2020)
AgNPs from *Lysiloma acapulcensis*	Green synthesis using *L. acapulcensis* extract	Spherical and quasi-spherical	1.2–62	0.1–5 µg/mL for chemical nanoparticles and 0.02–1 µg/mL for biogenic nanoparticles for broth microdilution assay	*C. albicans, E. coli, S. aureus, P. aeruginosa*	Disk diffusion and broth microdilution assays	Not determined	Not determined	Garibo et al. (2020)
AgNPs from *Penicillium oxalicum*	Biogenic AgNPs derived from fungal metabolites	Spherical	60–80	5/10, 10/20, 15/30, and 20/40 μg/μl of the AgNPs dilutions of 1, 3, and 5 mM for Agar well diffusion and 1, 3, and 5 mM for broth dilution assays	*S. aureus, S. dysenteriae, S.* Typhi	Agar well diffusion, broth dilution assays	Not determined	Not determined	Feroze et al. (2020)
AgNPs from *Padina* spp.	Phytosynthesis using marine algae extract (*Padina* spp.)	Spherical and oval-shaped while some observed to be irregular-shaped and polydis-persed	25–60	0.25 mg/ml, 0.50 mg/ml, 0.75 mg/ml, and 1.00 mg/ml	*S. aureus, B. subtilis, P. aeruginosa, S.* Typhi*, E. coli*	Disk diffusion	Not determined	Not determined	Bhuyar et al. (2020)
AgNPs from *Aloe vera*	Green synthesis using *Aloe vera* extract	Not specified	Not specified	Not determined	*E. coli, P. aeruginosa, Enterobacter* spp.*, S. aureus*	Disk diffusion	Not determined	Not determined	Anju et al. (2020)
MOF-AgNPs	AgNPs synthesized using *Moringa oleifera* flower extract	Spherical	8	Not determined	*K. pneumoniae, S. aureus*	Disk diffusion	Not determined	Not determined	Bindhu et al. (2020)
AgNPs from *Bacillus subtilis*	Biosynthesis using *B. subtilis* isolates	Spherical, hexagonal, and irregular	20	21–170 mg/ mL	*E. coli, S. aureus, P. aeruginosa, B. cereus, S.* Typhi, *Candida albicans*	Broth dilution	Not determined	Not determined	El-Bendary et al. (2020)
AgNPs from *Gelidium corneum*	Phytosynthesis via marine red algae extract	Spherical	20–50	0.08 -32.7 μg /ml	*E. coli*	Broth dilution	Damage to membrane and cell wall	Transmission electron microscopy	Yılmaz Öztürk et al. (2020)
AgNPs from *Shewanella* spp. ARY1	Biogenic synthesis from *Shewanella* culture supernatant	Spherical	38	20 μL of different concentrations (20, 30 and 40 μg/mL) for disk diffusion	*E. coli, K. pneumoniae*	Disk diffusion and broth dilution assays	Cell lysis and membrane disruption	Transmission electron microscopy	Mondal et al. (2020)
SA-AgNPs, GL-AgNPs, BR-AgNPs	AgNPs biosynthesized from *Semecarpus anacardium, Glochidion lanceolarium, Bridelia retusa*	Spherical	62.72, 93.23, 74.56	Not determined	*P. aeruginosa, E. coli, S. aureus*	Broth dilution	Not determined	Not determined	Mohanta et al. (2020)
AgNPs from *Cestrum nocturnum*	Phytosynthesis using *C. nocturnum* extract	Spherical	20	0-256 μg/ml for broth dilution assays	*Citrobacter, E. faecalis, S.* Typhi*, E. coli, P. vulgaris, V. cholerae*	Disk diffusion and broth dilution	Not determined	Not determined	Keshari et al. (2020)
Cp-AgNPs	AgNPs synthesized using *Cucumis prophetarum* leaf extract	Polymorphic	30–50	20, 50, and 75 μg/ml	*S. aureus, S.* Typhi	Disk diffusion	Not determined	Not determined	Hemlata et al. (2020)
bAgNPs	Biogenic AgNPs from *Caesalpinia digyna*	Not specified	11.3–45.4	15, 30, and 60 μg for disk diffusion	*B. subtilis, E. coli* DH5α*, E. coli* K12, enteropathogenic *E. coli, S.* Typhi	Disk diffusion and broth dilution	Fatty acid oxidation, interaction with cellular macromolecules	Lipid peroxidation assay	Niloy et al. (2020)
AgNPs from *Phingobium* spp. MAH-11	Biogenic synthesis via *Phingobium* extract	Spherical	7–22	30 ul at 500 ppm and 1000 ppm for disk diffusion	*S. aureus, E. coli*	Disk diffusion, broth dilution	Irregular, wrinkled, deformed cell wall	Field emission scanning electron microscopy	Akter and Huq (2020)
AgNPs from *Nigella sativa, Piper nigrum* L.	Green synthesis using aqueous extracts	Spherical	20–50	Not determined	*B. megaterium, B. subtilis, S. aureus, E. coli, K. oxytoca, P. aeruginosa*	Disk diffusion	Not determined	Not determined	Mahfouz et al. (2020)
AgNPs from *Citrus limetta*	Phytosynthesis via *C. limetta* peel extract	Spherical	18	107 μg/mL for agar well diffusion and 4.28-107 μg/mL μg/mL for broth dilution assay	*M. luteus, S. mutans, S. epidermidis, S. aureus, E. coli*	Agar well diffusion, broth dilution	Anti-biofilm, membrane permeabilization, morphological deformities	SEM analysis	Dutta et al. (2020)
AgNPs from *Berberis vulgaris*	Green synthesis using leaf and root extracts (*Berberis vulgaris*)	Spherical	30–70	1, 3, 5 mM nanoparticles for broth dilution assay	*E. coli, S. aureus*	Disk diffusion, broth dilution	Not determined	Not determined	Behravan et al. (2019)
AgNPs from *Sapindus mukorossi*	Biogenic synthesis via *S. mukorossi* fruit extract	Spherical	17.3	60, 30, and 15 μg/mL	*P. aeruginosa, S. aureus*	Agar disc and agar well-diffusion methods	Not determined	Not determined	Huong and Nguyen (2019)
OV-AgNPs	AgNPs derived from *Origanum vulgare L.*	Spherical	2–25	Not determined	*E. coli, P. aeruginosa, S.* Typhi*, S. sonnei, M. luteus, S. epidermidis, MRSA, S. aureus, A. flavus, P. alba, P. variotii*	Well diffusion assay	Not determined	Not determined	Shaik et al. (2018)
AgNPs from *Azadirachta indica*	Green synthesis using *A. indica* extract	Spherical	65	2, 4, 8, 16 μg/mL	*P. aeruginosa*	Disk diffusion	Not determined	Not determined	Senthilkumar et al. (2018)
AgNPs from *Punica granatum*	Phytosynthesis using *P. granatum* bark extract	Spherical	20–40	25, 50, 75, and 100 μL	*E. coli, P. aeruginosa, P. vulgaris, S.* Typhi*, S. aureus, S. epidermidis, K. pneumoniae*	Well diffusion assay	Not determined	Not determined	Devanesan et al. (2018)
AgNPs	Commercial 10 nm AgNPs	Not determined	10	Different concentrations of 10 nm AgNPs (5.0, 1.25, and 0.156 µg/mL)	*P. aeruginosa*	Time Killing assay	Not determined	Not determined	Salomoni et al., 2017

### 4.1 Synthesis and broad-spectrum antibacterial activity

AgNPs have been widely studied for their antibacterial properties. Green-synthesized AgNPs using *Teucrium polium* leaf extract exhibited strong antimicrobial effects against multiple bacterial strains, including *Staphylococcus aureus*, *Bacillus subtilis*, *E. coli*, *Klebsiella pneumoniae*, and *Pseudomonas aeruginosa*. The antimicrobial activity was evaluated using both disk diffusion and broth dilution assays, showing significant inhibition of bacterial growth (Aljowaie and Aziz, 2025). Similarly, AgNPs synthesized from *Teucrium Parvifolium* seeds demonstrated high efficacy against *E. coli O157:H7*, *Enterococcus faecalis*, *P. aeruginosa*, and *S. aureus* using similar evaluation methods (Soltani et al., 2024).

Plant extracts have been widely documented for their diverse therapeutic applications (Abd El-Hafeez et al., 2018, 2022). Green-synthesized AgNPs using various plant extracts have shown significant antibacterial properties (**Table 1**). AgNPs derived from *Olive leaf wastes* exhibited notable activity against *Listeria monocytogenes*, *Bacillus cereus*, *S. aureus*, *E. coli*, *Yersinia enterocolitica*, and *Campylobacter jejuni* (Alowaiesh et al., 2023). Similarly, AgNPs synthesized from *Argyreia nervosa* leaf extract effectively inhibited enteropathogenic *E. coli* (EPEC) (Parvathalu et al., 2023). AgNPs derived from *Citrus limon* zest extract demonstrated inhibitory effects against *S. aureus*, *E. coli*, and *C. albicans* via disk diffusion assays (Khane et al., 2022). Furthermore, *Gardenia thailandica*-synthesized AgNPs exhibited strong antibacterial activity against *S. aureus*, as confirmed by both disk diffusion and *in vivo* antibacterial studies in rats (Attallah et al., 2022). The efficacy of phytosynthesized AgNPs was further highlighted in a study by Hajizadeh et al. (2024), where AgNPs synthesized from *Lepidium draba* leaves exhibited potent antimicrobial activity against *E. coli*, *K. pneumoniae*, *S. aureus*, *E. faecalis*, and *Candida albicans*.

Biosynthesized AgNPs using bacterial strains have also shown effective antimicrobial action. AgNPs derived from *Lactobacillus* and *Bacillus* species demonstrated strong inhibition against *P. aeruginosa* and *S. aureus*, as confirmed by disk diffusion assays at concentrations of 10, 20, and 40 μg/mL (Al-Asbahi et al., 2024). Furthermore, Basheer et al. (2023) reported the biosynthesis of AgNPs using marine fungi (*Penicillium simplicissimum*, *Aspergillus terreus*, *A. japonicus*, and *A. oryzae*), which displayed significant antimicrobial effects against *E. coli*, *K. pneumoniae*, *P. vulgaris*, *S.* Typhi, *E. faecalis*, *S. aureus* (methicillin-resistant *S. aureus* (MRSA)), *S. hominis*, and *S. epidermidis* using the agar well diffusion assay at 2, 5, and 8 mM concentrations. Similarly, Shumi et al. (2023) synthesized AgNPs from *Lippia abyssinica* plant extract, which exhibited antimicrobial activity against *S. aureus* and *E. coli* at a concentration of 62.5 μg/mL using the agar well diffusion method.

AgNPs produced via chemical synthesis have also been assessed for their antibacterial potency (**Table 1**). For instance, Baveloni et al. (2025) synthesized spherical AgNPs (~24.3 nm) that exhibited significant antimicrobial activity against *S. aureus*, *P. aeruginosa*, and *E. coli* using the broth microdilution assay at concentrations ranging from 6.74 to 117 μg/mL. Similarly, Iwuji et al. (2024) reported chemically synthesized AgNPs (~58.3 nm) demonstrating potent inhibition against the same bacterial species, further confirming their broad-spectrum efficacy.

### 4.2 Enhanced antibacterial efficacy through nanoparticle modifications

Nanoparticle modifications have been explored to enhance antimicrobial properties. AgNPs conjugated with polyethylene glycol (PEG) and nystatin (AgNPs-PEG-NYS) exhibited superior antibacterial activity against *S. aureus* and *E. coli* compared to non-functionalized AgNPs, as evidenced by agar well diffusion assays (Ibraheem et al., 2024). Furthermore, AgNPs integrated with catecholamine-based polymers (PDA) displayed notable antibacterial activity against *E. coli* (Caniglia et al., 2024). Caniglia et al. (2024) synthesized AgNPs incorporated into catecholamine-based polymers (AgNPs-PDA) using a double potentiostatic method, which exhibited significant structural modifications in *E. coli* membranes, leading to bacterial inhibition. Additionally, Balciunaitiene et al. (2024) developed a polymer film embedded with biosynthesized AgNPs using *Symphyti radix* root extracts, demonstrating strong antibacterial activity against *S. aureus*, β-hemolytic *streptococcus*, *S. epidermidis*, *E. faecalis*, *E. coli*, *K. pneumoniae*, *P. aeruginosa*, *P. vulgaris*, *B. cereus*, and *C. albicans*.

### 4.3 Fungal inhibition

Apart from their efficacy against bacteria, AgNPs have exhibited antifungal activity (**Table 1**). AgNPs synthesized using *Lepidium draba* L. leaves demonstrated strong inhibitory effects against *C. albicans* (Hajizadeh et al., 2024). Furthermore, Tunç (2024) reported significant antifungal activity of chemically synthesized AgNPs and carboplatin-loaded AgNPs (AgNPs-Car) against *C. albicans* and *C. tropicalis* using broth microdilution assays. Similarly, Balciunaitiene et al. (2024) showed inhibition of *C. albicans* by AgNPs incorporated into a natural polymer film biosynthesized with *Symphyti radix* extract. In another green synthesis approach, Cao et al. (2021) evaluated lignin-stabilized AgNPs (L-AgNPs) and observed inhibition of *C. albicans* growth via disk diffusion. Broader antifungal activity was noted by Hasanin et al. (2021), who reported activity of AgNP-based nanocomposites (Ag-NC) against multiple fungal strains including *C. albicans*, *A. niger*, *A. terreus*, *A. flavus*, and *A. fumigatus*. Additionally, Renganathan et al. (2021) identified antifungal activity of AgNPs from *Bauhinia tomentosa* against *C. albicans* via molecular docking, indicating potential interactions with fungal protein targets. This may be indicated as an efficient approach develop new therapies to combat the emerging antifungal resistance (Khalifa et al., 2024a; Khalifa et al., 2024c).

## 5 Antimicrobial mechanisms of AgNPs

**Figure 2:**
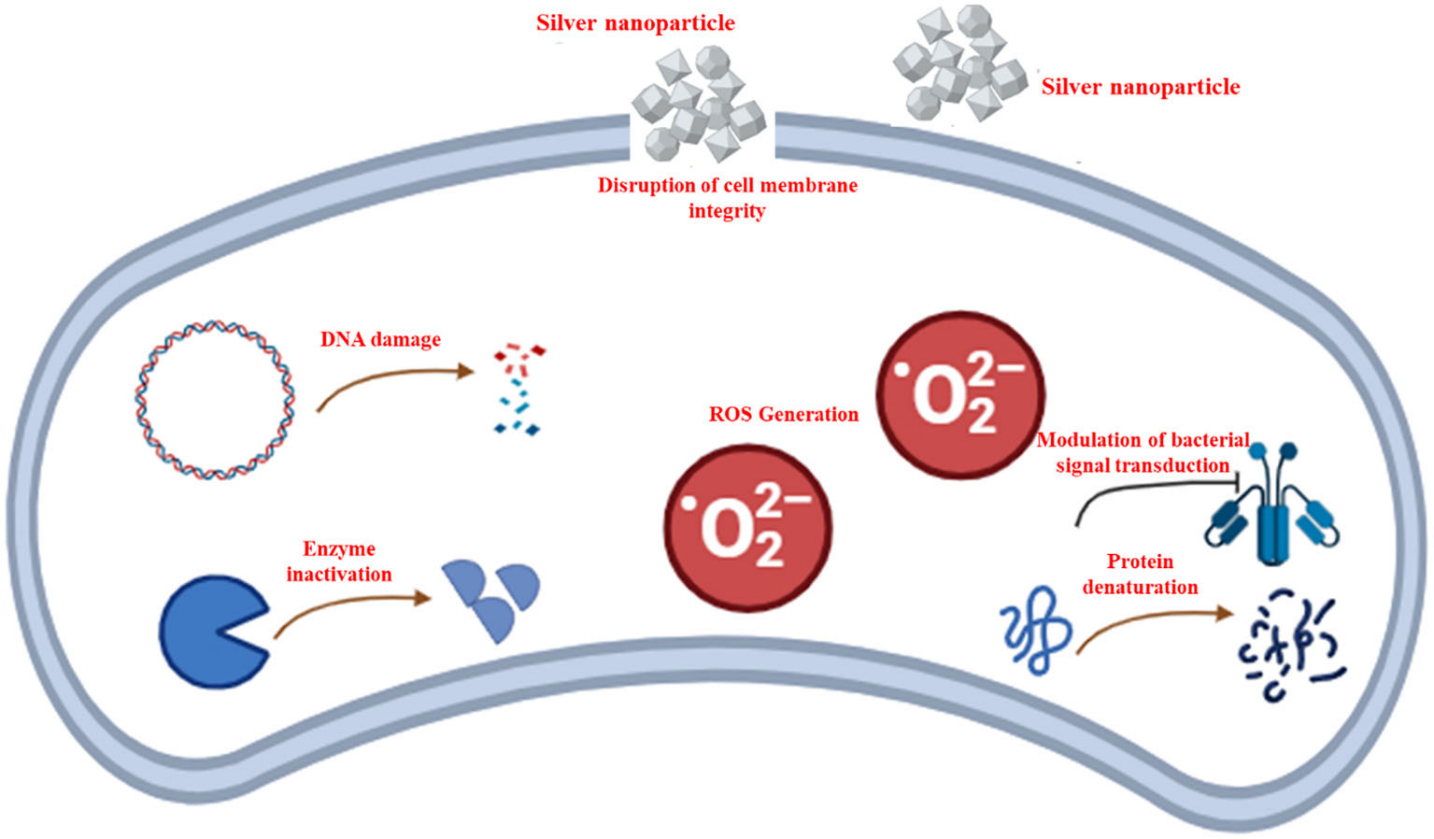
A schematic representation illustrating the antibacterial mechanisms of silver nanoparticles (AgNPs), highlighting the disruption of cell membrane integrity, reactive oxygen species (ROS)-dependent pathway, DNA damage, protein denaturation, enzyme inactivation, and modulation of bacterial signal transduction.

The capacity of AgNPs to inhibit microbial growth has been thoroughly investigated, with ongoing efforts to elucidate the underlying processes. Research indicates that several mechanisms are associated with AgNPs. It can attach to and subsequently breach bacterial cell walls, leading to cell membrane damage and the release of internal components (Dakal et al., 2016). Moreover, AgNPs can disrupt vital internal cellular functions, such as interfering with the respiratory pathway, disrupting DNA duplication, and halting cell proliferation (Wang et al., 2016). The processes by which AgNPs combat bacteria are depicted in **Figure 2**. In this section, we will discuss the major mechanisms associated with the antimicrobial effects of AgNPs.

### 5.1 Disruption of bacterial cell membrane integrity

One of the primary antimicrobial mechanisms of AgNPs involves their interaction with bacterial cell walls and membranes. AgNPs exhibit strong affinities for bacterial membranes due to their positive charge, which facilitates electrostatic interactions with negatively charged bacterial surfaces (Mikhailova, 2024). Upon attachment, AgNPs can cause structural disintegration of the cell membrane, leading to increased permeability and leakage of cytoplasmic contents. This process ultimately compromises bacterial viability and initiates cell death (Dakal et al., 2016). The antimicrobial efficacy of AgNPs is significantly influenced by the structural differences in bacterial cell walls. Gram-negative bacteria, such as *E. coli*, tend to be more vulnerable to AgNPs than Gram-positive bacteria like *S. aureus*. This is primarily due to variations in peptidoglycan composition and thickness. Gram-positive bacteria possess a substantially thicker peptidoglycan layer (~30 nm), which serves as a protective barrier, whereas Gram-negative bacteria have a thinner peptidoglycan layer (~3–4 nm) (Rai et al., 2012). The negatively charged peptidoglycan in Gram-positive bacteria can also bind silver ions, limiting their penetration and reducing their antimicrobial effectiveness (Feng et al., 2000). Conversely, Gram-negative bacteria are more susceptible to AgNPs due to their thinner cell wall and the presence of lipopolysaccharides (LPS), which not only contribute to membrane stability but also facilitate AgNP adhesion through electrostatic interactions. This enhanced attachment leads to greater bacterial inhibition, even at lower AgNP concentrations (Pal et al., 2007). Several studies confirm that AgNPs preferentially accumulate on the surface of Gram-negative bacteria due to LPS, increasing their antimicrobial susceptibility (Pal et al., 2007). These structural and compositional differences explain why *S. aureus* exhibits greater resistance, while *E. coli* is significantly inhibited by AgNPs, establishing a clear relationship between AgNP concentration and bacterial cell wall properties (Dakal et al., 2016).

### 5.2 Generation of ROS

AgNPs have been shown to induce oxidative stress in bacterial cells by generating ROS, including hydroxyl radicals (^•^OH), superoxide anions (O_2_^•−^), and hydrogen peroxide (H_2_O_2_) (He et al., 2011). These ROS are highly reactive and can cause significant cellular damage by oxidizing lipids, proteins, and DNA. Silver ions (Ag^+^) interfere with the function of the respiratory electron transport chain by inhibiting key respiratory enzymes, leading to its uncoupling from oxidative phosphorylation. This disruption affects the efficiency of cellular respiration, ultimately impairing energy production (Holt and Bard, 2005; Dakal et al., 2016). The excessive accumulation of free radicals resulting from this process causes direct oxidative damage to the mitochondrial membrane, inducing necrosis and ultimately leading to cell death. Additionally, increased ROS levels contribute to the oxidation of essential biomolecules, including lipids, proteins, and DNA, further exacerbating cellular damage (Juan et al., 2021). Free radicals also interact with lipid molecules, which are abundant in cellular membranes, triggering lipid peroxidation. This process generates lipid hydroperoxides as an initial step in ROS formation, particularly affecting polyunsaturated fatty acids (Chandimali et al., 2025). AgNP-mediated ROS production also affects the activity of various antioxidant enzymes, including NADPH-dependent flavoenzyme, catalase, glutathione peroxidase, and superoxide dismutase, disrupting the balance between ROS generation and detoxification (Dakal et al., 2016).

### 5.3 Interaction with intracellular components and processes

Following penetration of the bacterial cell, AgNPs interfere with various intracellular processes. They can bind to essential enzymes and proteins, leading to the inhibition of critical metabolic pathways (More et al., 2023). Additionally, AgNPs can displace essential metal ions, such as zinc and iron, from bacterial proteins, thereby disrupting enzyme functions and cellular homeostasis (Girma, 2023).

Another critical antimicrobial mechanism of AgNPs is their ability to interact with bacterial nucleic acids. AgNPs can directly bind to bacterial DNA, causing structural distortions that hinder replication and transcription. Ag^+^ ions intercalate between purine and pyrimidine base pairs, disrupting the hydrogen bonds between the complementary DNA strands and thereby destabilizing the double-helix structure (Klueh et al., 2000). Additionally, AgNPs induce structural changes in DNA, causing it to transition from a relaxed to a condensed state, which ultimately inhibits its ability to replicate (Feng et al., 2000).

Furthermore, Ag^+^ has been shown to interact with functional groups in proteins, leading to their deactivation. Specifically, Ag^+^ ions bind to thiol (-SH) groups in membrane-associated proteins, forming stable Ag–S bonds that disrupt protein function (Fahim et al., 2024). These proteins play essential roles in transmembrane ATP production and ion transport across the cell membrane (Klueh et al., 2000). Both AgNPs and Ag^+^ ions can alter the three-dimensional structure of proteins, disrupt disulfide bonds, and block active binding sites, ultimately impairing bacterial proliferation and contribute to AgNP-mediated cytotoxicity (Lok et al., 2006).

### 5.4 Modulation of bacterial signaling transduction pathways

Recent studies suggest that AgNPs can interfere with bacterial quorum sensing (QS) and signal transduction pathways (Aflakian and Hashemitabar, 2025). Quorum sensing is a critical communication mechanism that bacteria use to regulate gene expression and coordinate collective behaviors, including biofilm formation and virulence. AgNPs have been shown to inhibit quorum sensing by disrupting signaling molecules, thereby preventing the establishment of biofilms and reducing bacterial pathogenicity (Awadelkareem et al., 2023). Studies have demonstrated that AgNPs serve as effective anti-QS agents, inhibiting biofilm formation and reducing violacein production in *Chromobacterium violaceum* (Jagtap and Priolkar, 2013). Additionally, green-synthesized AgNPs have shown significant potential in managing microbial infections. Research indicates that AgNPs can interfere with the synthesis of QS signaling molecules by inhibiting the LasI and RhlI synthases, thereby disrupting bacterial communication and virulence regulation (Lahiri et al., 2021). Furthermore, AgNPs have been shown to downregulate quorum sensing-related genes. In *P. aeruginosa*, green-synthesized AgNPs exhibited a dose-dependent inhibition of pyocyanin production, a key virulence factor (Selem et al., 2022). Pyocyanin, a blue redox-active secondary metabolite, plays a crucial role in biofilm development and significantly contributes to bacterial evasion of the host immune system. By suppressing pyocyanin synthesis, AgNPs can weaken bacterial pathogenicity and enhance susceptibility to antimicrobial treatments (Awadelkareem et al., 2023). Beyond quorum sensing inhibition, AgNPs can also modulate bacterial signal transduction pathways by interfering with phosphorylation-based signaling cascades (More et al., 2023). Many bacterial regulatory systems rely on histidine kinases and response regulators to sense environmental changes and control adaptation (Capra and Laub, 2012). Furthermore, analyzing the phosphotyrosine profile of bacterial proteins in both Gram-positive and Gram-negative bacteria provides valuable insight into how AgNPs influence bacterial signal transduction pathways. These pathways regulate essential cellular functions, including growth and metabolism. The reversible phosphorylation of tyrosine residues in key protein substrates, such as RNA polymerase sigma factor (RNA pol σ factor), single-stranded DNA binding proteins (ssDBPs), and UDP-glucose dehydrogenase, is crucial for their activation (Mijakovic et al., 2006). Once phosphorylated, these proteins play significant roles in DNA replication, recombination, metabolism, and cell cycle regulation. Consequently, AgNP-mediated inhibition of protein phosphorylation disrupts enzymatic activity, ultimately hindering bacterial growth and survival (Dakal et al., 2016).

### 5.5 Induction of apoptotic-like cell death

In addition to oxidative stress and metabolic disruption, AgNPs can trigger apoptosis-like responses in bacteria. Some studies have demonstrated that AgNPs activate bacterial self-destruction pathways, akin to programmed cell death in eukaryotic cells. Research has demonstrated that AgNPs can inhibit the growth of *E. coli* and trigger apoptosis-like cell death (Kim and Lee, 2021). However, the exact mechanism underlying AgNP-induced apoptosis-like death, as well as its potential link to DNA damage-inducible protein F (DinF), a key component of the SOS response, remains unclear (Kim and Lee, 2021).

## 6 Antimicrobial resistance to AgNPs

**Figure 3:**
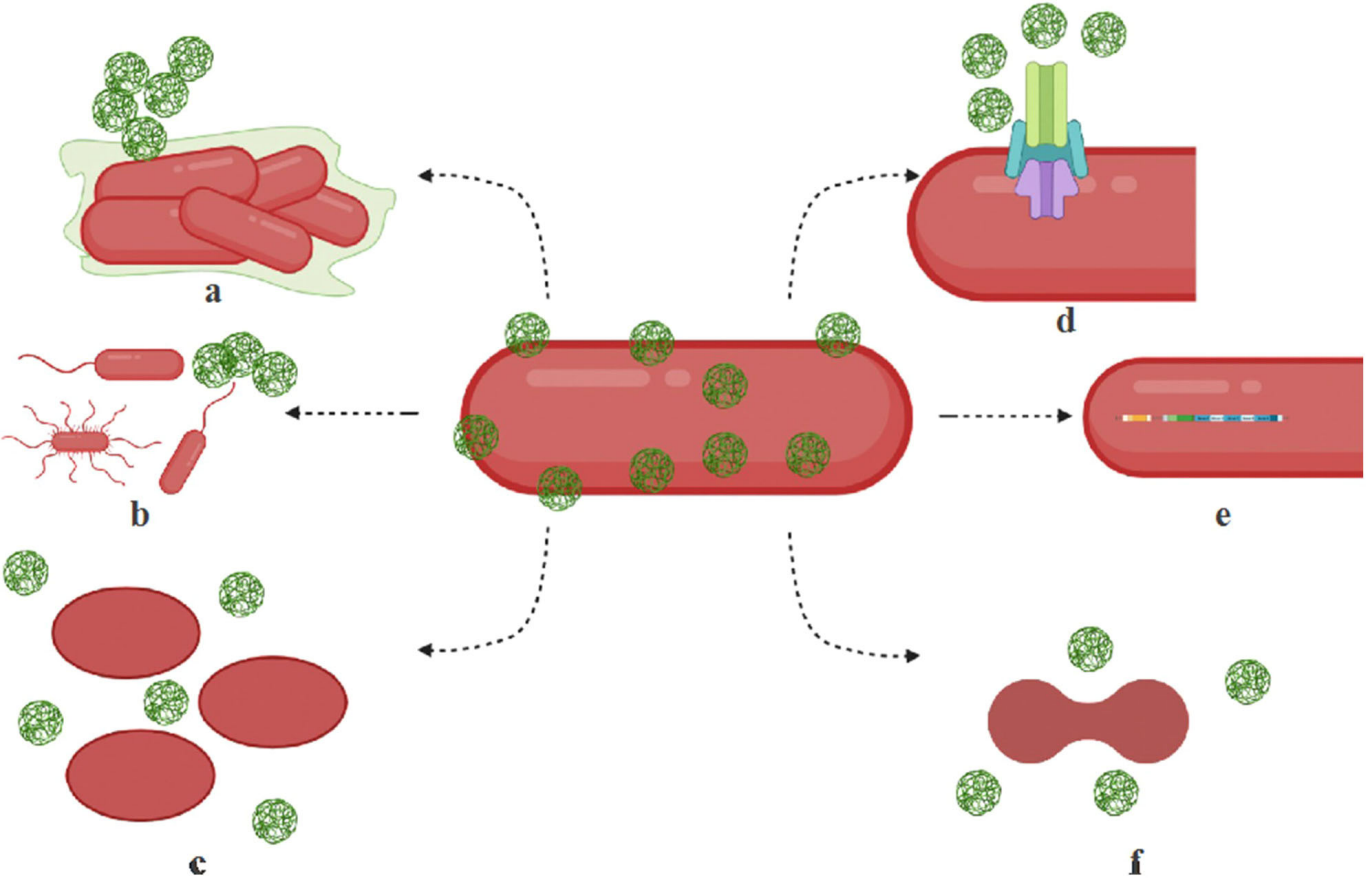
Mechanisms of microbial resistance to nanoparticles. Microorganisms employ various strategies to resist the effects of nanoparticles. **(a)** Biofilm formation occurs when bacteria produce exopolysaccharides that create a protective biofilm or facilitate nanoparticle aggregation. **(b)** Motility adaptations enable hypermotile bacteria to evade nanoparticles and optimize nutrient uptake. **(c)** Morphological alterations allow bacteria to change their shape, such as transitioning from rod-shaped to oval forms, through modifications in fatty acids, membrane lipids, and proteins, helping them filter out nanoparticles. **(d)** Efflux systems contribute to resistance by overexpressing efflux pump complexes that actively expel nanoparticles from bacterial cells. **(e)** Operon activation plays a role in resistance by triggering cytoprotective mechanisms through specialized operons and resistance genes. **(f)** Cell division interference occurs when nanoparticle-induced stress disrupts cell cycle regulation, further enhancing microbial resistance. These mechanisms collectively allow bacteria to withstand nanoparticle exposure, posing challenges for antimicrobial treatments. Reproduced with permission from (Kamat and Kumari, 2023), under License CC BY 4.0.

Microbial resistance to nanoparticles develops through various adaptive mechanisms, including efflux pumps, biofilm formation, exopolysaccharide overproduction, genetic mutations, and metabolic alterations as shown in **Table 2**, **Figure 3** (Kamat and Kumari, 2023). AgNPs are among the most widely used nanomaterials in commercial products, particularly in personal care items. Consequently, it is not surprising that bacteria have developed resistance to them. Studies have demonstrated that chronic exposure to AgNPs leads to the emergence of bacterial resistance. For example, *E. coli* K-12 MG1655 exhibited resistance to citrate-coated AgNPs after 225 generations, linked to mutations in *cusS, purI, rpoB*, and *ompR* (Graves et al., 2015). Similarly, *E. coli* BW25113 ΔyhaK developed resistance through the overproduction of exopolysaccharides, which likely hinder nanoparticle penetration (Joshi et al., 2012). Another notable adaptation is the production of flagellin, observed in *E. coli* O13 and *P. aeruginosa* CCM 3955, which promotes nanoparticle aggregation and reduces their antimicrobial effectiveness (Panáček et al., 2018). Additionally, prolonged exposure to silver sulfide-coated nanoparticles in *E. coli* resulted in the upregulation of MDR genes and copper efflux transporters, further enhancing bacterial survival under nanoparticle stress (Li et al., 2019). Furthermore, McNeilly et al. (2021) reported that the prolonged use of AgNO_3_ and AgNPs against *E. coli* led to the development of resistance to Ag^+^, driven by the induction of endogenous mutations.

**Table 2:** Common resistance mechanisms to silver nanoparticles.

Type of Nanoparticle	Resistant Microorganism	Resistance Emergence (Generations/Days)	Observed Genetic, Cellular, or Phenotypic Adaptations	Reference
Citrate-coated silver nanoparticles	*E. coli* K-12 MG1655	225 generations	Mutations identified in *cusS, purI, rpoB, ompR*	Graves et al., 2015
Silver nanoparticles	*E. coli* BW25113 ΔyhaK	Not specified	Increased exopolysaccharide production	Joshi et al., 2012
Silver nanoparticles	*E. coli* O13, *P. aeruginosa* CCM 3955, *E. coli* CCM 3954	Not specified	Adhesive flagellum protein (*flagellin*) production, leading to nanoparticle aggregation	Panáček et al., 2018
Silver sulfide-coated silver nanoparticles	*E. coli*	More than 200 days	Upregulation of multidrug resistance (MDR) genes and copper efflux transporter genes	Li et al., 2019
Silver nanoparticles	Environmental or clinical microbiota model (*E. coli* and *Bacillus* spp.)	Not specified	Altered Z-ring division septum formation, increased expression of cytoprotective genes, permease components, and efflux proteins	Gunawan et al., 2013

The environmental persistence of AgNPs also contributes to resistance development. In mixed microbiota models including *E. coli* and *Bacillus* species, exposure to AgNPs led to significant genetic and phenotypic changes, such as modifications in cell division machinery and upregulation of cytoprotective genes, permease components, and efflux proteins (Gunawan et al., 2013). Long-term presence of AgNPs in natural ecosystems raises concerns about their role in promoting co-selection of antibiotic resistance genes. Studies have reported that bacteria exposed to AgNPs can develop cross-resistance to multiple antibiotics, including penicillin, kanamycin, ciprofloxacin, and gentamicin (Li et al., 2019). This phenomenon is linked to oxidative stress responses that drive the overexpression of efflux pump genes such as *marA* and *acrAB-tolC*, enabling bacteria to expel both silver ions and antibiotics effectively. Additionally, silver-resistant *E. coli* strains have been found to carry resistance genes for multiple antibiotics, including beta-lactams (*blaCTX-M*), quinolones (*oqxAB*), and aminoglycosides (*aac-Ib-cr*) (Fang et al., 2016). Furthermore, environmental studies highlight the impact of AgNPs on microbial communities in soil and water. The release of nanosilver into these environments may lead to co-selection for antibiotic resistance determinants, increasing the persistence of resistant pathogens in nature (Pal et al., 2017). This underscores the importance of evaluating nanoparticle waste disposal and the long-term effects of nanosilver on microbial ecosystems.

## 7 Targeted delivery systems to enhance the antimicrobial efficacy of AgNPs

The application of targeted delivery systems has significantly enhanced the antimicrobial activity of AgNPs, particularly against MDR bacteria. Despite their well-established bactericidal properties, conventional AgNPs suffer from limitations such as non-specific interactions, rapid aggregation, and toxicity to mammalian cells (Liao et al., 2019; Ipe et al., 2020). These challenges necessitate the development of targeted strategies that can improve AgNP selectivity, stability, and controlled release while minimizing adverse effects. Advanced approaches, including surface functionalization, biopolymer encapsulation, liposomal carriers, stimuli-responsive systems, and antibody-conjugated AgNPs, have been extensively explored to optimize AgNP delivery and enhance their therapeutic potential (**Table 3**).

**Table 3:** Targeted delivery systems for enhanced AgNP antimicrobial efficacy.

Delivery Strategy	Mechanism	Targeted Pathogen	Reference
Surface functionalization	Functionalized with chitosan for enhanced bacterial adhesion and mucoadhesion	*E. coli*, MRSA, *S. aureus*	Krishnaraj et al., 2022; Peng et al., 2017
Biopolymer encapsulation	Encapsulated within alginate, PLGA, and gelatin for controlled silver ion release	*P. aeruginosa*, *S. aureus*, *S. pyogenes*	Stevanović et al., 2012; Srichaiyapol et al., 2022
Liposomal carriers	Liposomal encapsulation enhances bioavailability and prevents premature degradation	*E. coli*, *P. aeruginosa*, *S. aureus*	Kumar et al., 2023; Eid and Azzazy, 2014
Stimuli-responsive systems	pH-sensitive hydrogels trigger AgNP release in response to bacterial microenvironment	Gram-positive and Gram-negative wound pathogens	Haidari et al., 2021
Enzyme-responsive systems	ANAs collapse in response to SplB enzyme activity, increasing MRSA targeting	MRSA	Zuo et al., 2020
Antibody-conjugated AgNPs	Functionalized with bacterial-specific antibodies for precision targeting	*S. aureus*, Gram-negative bacteria	Al-Sharqi et al., 2020; Ramírez Saenz et al., 2024

### 7.1 Surface functionalization for enhanced targeting

One of the most effective methods for improving AgNP targeting is surface functionalization with biocompatible ligands, which facilitates selective bacterial adhesion and penetration (Fu et al., 2024). Among the various functionalization approaches, chitosan-coated AgNPs have demonstrated superior mucoadhesive properties, allowing for stronger electrostatic interactions with negatively charged bacterial membranes (Krishnaraj et al., 2022). For instance, Wang et al. developed a chitosan/oxidized konjac glucomannan hydrogel incorporating AgNPs for the treatment of irregular wounds. The hydrogel exhibited self-healing properties, strong tissue adhesion, and potent antibacterial activity (Wang et al., 2020). Another example is chitosan-coated AgNPs, which have proven effective in treating wounds infected with MRSA (Peng et al., 2017). Furthermore, research by Mostafa et al. (2022) demonstrated that chitosan-silver conjugates exhibit promising broad-spectrum anti-biofilm activity against *B. subtilis*, *P. aeruginosa*, *S. aureus*, and *E. coli* (Mostafa et al., 2022). Similarly, AgNPs conjugated with antimicrobial peptides (AMPs), such as LL-37, help overcome their inherent limitations. This combination shows promise as a potential therapeutic agent against antibiotic-resistant bacteria, particularly MRSA (Masimen et al., 2022). Additionally, functionalizing AgNPs with folic acid has demonstrated promising antibacterial activity against both Gram-negative (*E. coli*) and Gram-positive (*S. aureus*) bacteria (Chowdhuri et al., 2015). These modifications enable AgNPs to achieve higher bacterial selectivity while reducing unintended cytotoxicity to human cells.

### 7.2 Biopolymer encapsulation for controlled release

Encapsulation within biodegradable polymeric matrices represents another promising approach for controlled AgNP release and prolonged antimicrobial effects. Natural and synthetic biopolymers, including alginate, poly(lactic-co-glycolic acid) (PLGA), and gelatin, have been utilized as nanocarriers to enhance AgNP stability and mitigate toxicity. For example, a hydrogel incorporating tannic acid-stabilized AgNPs (TA-AgNPs/alginate) exhibited strong antibacterial activity against *S. pyogenes, S. aureus*, and *P. aeruginosa.* Additionally, it showed promising potential for treating complex wound biofilms (Srichaiyapol et al., 2022). Rugaie et al. (2022) developed a straightforward, single-step method to coat AgNPs using polymeric stabilizers, specifically polyvinylpyrrolidone (PVP) and ethyl cellulose (EC) (Rugaie et al., 2022). Their investigation demonstrated that these coated AgNPs effectively inhibited biofilm formation by clinical isolates of *E. coli* on urinary catheters. Notably, AgNPs coated with PVP exhibited significantly greater biofilm inhibition compared to those stabilized with EC. Furthermore, PLGA-encapsulated AgNPs ensure a controlled and sustained release of silver ions, offering prolonged and enhanced antimicrobial activity while reducing host toxicity (Stevanović et al., 2012). Moreover, gelatin-PVA-AgNPs hydrogel has been explored for wound healing applications, demonstrating accelerated tissue regeneration while maintaining potent antimicrobial activity (Bag et al., 2022). The incorporation of AgNPs into polymeric matrices not only enhances their therapeutic efficacy but also facilitates localized drug delivery, thereby reducing systemic toxicity.

### 7.3 Liposomal carriers for improved bioavailability

Liposomal carriers have emerged as effective nanocarriers for improving AgNP bioavailability and stability. Liposomal encapsulation shields AgNPs from premature degradation and enhances their circulation time in the biological environment. Liposomes are spherical vesicles with a phospholipid bilayer capable of encapsulating various chemical compounds (Kumar et al., 2023). Their unique bilayer structure enables the efficient entrapment of both hydrophilic and hydrophobic drugs, making them a versatile and promising platform for drug delivery applications (Kumar et al., 2023). Previous research has shown nanoliposomes loaded with AgNPs exhibit potent broad-spectrum antimicrobial activity against various pathogens, including *E. coli, S. enterica, P. aeruginosa*, and *S. aureus* (Eid and Azzazy, 2014). Additionally, these formulations have shown potential in promoting wound healing. This finding is reinforced by studies indicating that encapsulating antimicrobial agents, such as AgNPs, within nanoliposomes enhances their stability and targeted delivery. Additionally, nanoliposomes have demonstrated the ability to transport encapsulated agents directly to target bacteria in both *in vitro* and *in vivo* settings (Mozafari et al., 2021). The use of liposomal nanocarriers not only enhances AgNP stability but also reduces toxicity by preventing direct interaction with mammalian cells.

### 7.4 Stimuli-responsive AgNP delivery systems

In addition to passive targeting mechanisms, stimuli-responsive AgNP delivery systems offer an advanced strategy for spatiotemporal control over silver ion release. These systems are designed to respond to specific bacterial microenvironmental cues, such as pH variations, enzymatic activity, or oxidative stress levels. For example, a pH-responsive hydrogel has been developed to enable the controlled, pH-triggered release of AgNPs. This system is designed to detect changes in environmental pH and release AgNPs when the pH shifts from acidic to alkaline, a condition associated with pathogenic bacterial presence in wounds (Haidari et al., 2021). This innovative hydrogel shows promise as an effective material for treating infected wounds, demonstrating the ability to eliminate both Gram-negative and Gram-positive bacteria without causing toxicity to mammalian skin cells. Additionally, enzyme-responsive silver nanoparticle assemblies (ANAs) have been developed to selectively target MRSA (Zuo et al., 2020). These assemblies undergo a stable-to-collapsed transition upon encountering MRSA due to the decomposition of branched copolymers—used as macrotemplates in ANA synthesis—triggered by serine protease-like B (SplB) enzyme proteins. This structural transition significantly enhances the targeting affinity and efficiency of ANAs against MRSA. These smart nanoplatforms allow for on-demand silver release, minimizing toxicity while maximizing antibacterial efficacy.

### 7.5 Antibody-conjugated AgNPs for bacteria-specific targeting

A highly specific approach to AgNP targeting involves antibody-conjugated AgNPs, which are engineered to selectively bind to bacterial surface markers. This strategy enables highly targeted antimicrobial action while reducing off-target toxicity. For example, AgNPs functionalized with a specific antibody can be combined with laser radiation as an innovative treatment to selectively target resistant bacteria, particularly *S. aureus*, while minimizing effects on the normal microflora (Al-Sharqi et al., 2020). Similarly, AgNPs conjugated with BK510Lys endolysin at a concentration of 0.01 mg/mL, in a 2:1 ratio, at 40°C, and pH 5, exhibited a stronger inhibitory effect than AgNPs alone (0.5 µg/mL) against over 65% of the Gram-negative bacteria tested, indicating it highly specific alternative drugs for super-resistant Gram-negative bacteria (Ramírez Saenz et al., 2024). By harnessing the high specificity of monoclonal antibodies, antibody-functionalized AgNPs hold great potential for precision antimicrobial therapy against MDR pathogens.

## 8 Future directions

AgNPs have emerged as promising antimicrobial agents MDR bacteria. However, to maximize their therapeutic potential and overcome current limitations, several future directions should be considered. These approaches focus on enhancing efficacy, reducing toxicity, improving stability, and preventing bacterial resistance development.

### 8.1 Surface functionalization and conjugation

Enhancing the antimicrobial activity of AgNPs can be achieved by functionalizing their surface with bioactive molecules, polymers, or targeting ligands. For instance, conjugating AgNPs with antimicrobial peptides, antibodies, or small molecules can improve specificity and reduce non-specific interactions (Al-Sharqi et al., 2020; Ramírez Saenz et al., 2024). Additionally, coating AgNPs with biocompatible polymers such as PEG can enhance stability and bioavailability while reducing toxicity (Srichaiyapol et al., 2022; Ibraheem et al., 2024).

### 8.2 Synergistic combinations with antibiotics and natural compounds

Combining AgNPs with conventional antibiotics or natural antimicrobial agents may enhance their efficacy and prevent resistance development. Studies have shown that AgNPs can potentiate the effects of antibiotics by disrupting bacterial membranes and increasing drug uptake (Dove et al., 2023; More et al., 2023; Palau et al., 2023). For instance, recent studies have demonstrated that AgNPs conjugated with antibiotics such as amikacin (e.g., AgNPs_mPEG_AK) displayed enhanced antibacterial activity against MDR strains, including *E. coli*, *K. pneumoniae*, *P. aeruginosa*, and *A. baumannii* (Palau et al., 2023). These hybrid nanomaterials achieved notable activity at lower antibiotic concentrations, suggesting a dose-sparing effect. Moreover, integrating AgNPs with plant-derived bioactive compounds, such as flavonoids and essential oils, could provide a dual mechanism of action, improving antimicrobial potency and reducing cytotoxicity (Xu et al., 2020). Additionally, AgNPs synthesized using plant extracts—such as those from *Teucrium polium*, *Teucrium parvifolium*, *Lepidium draba L.*, and *Moringa oleifera*—have shown synergistic antimicrobial activity when paired with natural compounds like flavonoids, polyphenols, and essential oils. These green-synthesized AgNPs offer dual mechanisms: physical disruption of bacterial membranes and bioactive-mediated interference in bacterial metabolism, while also exhibiting reduced cytotoxicity compared to chemically synthesized counterparts (Aljowaie and Aziz, 2025; Soltani et al., 2024; Alowaiesh et al., 2023; Abdel-Rahman et al., 2022).

### 8.3 Controlled and targeted release systems

Developing advanced delivery systems, such as pH-responsive, enzyme-triggered, or temperature-sensitive nanocarriers, can help achieve controlled and targeted release of AgNPs. This approach can enhance antibacterial efficacy while minimizing exposure to healthy cells. For example, hydrogels or liposomes loaded with AgNPs have shown promising results in wound infections and biofilm-associated bacterial resistance (Mozafari et al., 2021; Wang et al., 2020). Additionally, nanocarriers designed for site-specific release can reduce the required dosage and mitigate potential cytotoxic effects.

### 8.4 Modulation of size, shape, and surface charge

The physicochemical properties of AgNPs, including size, shape, and surface charge, play a crucial role in their antimicrobial efficacy. Smaller nanoparticles exhibit greater surface area and enhanced bacterial interaction, while specific shapes, such as triangular or rod-shaped nanoparticles, have demonstrated improved antimicrobial effects compared to spherical ones (Liao et al., 2019; Rodrigues et al., 2024). Moreover, tuning the surface charge of AgNPs can influence their interaction with bacterial membranes, optimizing their antibacterial activity while reducing toxicity to mammalian cells (Bélteky et al., 2019).

### 8.5 Biosynthesis and green nanotechnology approaches

To improve the biocompatibility and environmental sustainability of AgNPs, green synthesis methods utilizing plant extracts, fungi, or bacteria have been explored. These eco-friendly approaches reduce the use of toxic chemical agents and enhance the biological properties of AgNPs (Zhang et al., 2016; Xu et al., 2020). Future research should focus on optimizing these biosynthetic techniques to ensure reproducibility, scalability, and clinical applicability.

### 8.6 Combating bacterial resistance to AgNPs

Although AgNPs exhibit broad-spectrum antimicrobial activity, there is a growing concern about bacterial adaptation and resistance. To mitigate this risk, researchers should investigate combination strategies, adaptive dosing regimens, and mechanisms to prevent bacterial efflux of silver ions. Additionally, integrating AgNPs with nanomaterials that disrupt bacterial communication systems, such as quorum sensing inhibitors, could reduce the likelihood of resistance development (Awadelkareem et al., 2023).

### 8.7 *In vivo* studies and clinical trials

Despite extensive *in vitro* research, the clinical translation of AgNP-based antimicrobials remains limited. Future studies should focus on *in vivo* models to evaluate pharmacokinetics, biodistribution, and long-term safety. Clinical trials are necessary to validate their effectiveness against MDR bacterial infections while assessing potential side effects (Zhang et al., 2016; Xu et al., 2020). Regulatory guidelines must also be established to ensure the safe application of AgNPs in medical and pharmaceutical settings.

## 9 Conclusion

In the era of emerging threats such as AMR, antifungal resistance, and global pandemics like COVID-19 (Khalifa and Al Ramahi, 2024), the search for effective infection therapies remains a critical challenge. Therefore, the development of alternative antimicrobial agents has become a priority in modern medicine (Khalifa et al., 2021b). AgNPs have demonstrated significant potential as next-generation antimicrobial agents due to their broad-spectrum antibacterial activity, unique physicochemical properties, and multiple mechanisms of bacterial inhibition. Their ability to disrupt bacterial membranes, interfere with essential biomolecules, and induce ROS production positions them as promising candidates for combating MDR bacterial infections.

Despite their advantages, concerns regarding bacterial adaptation, cytotoxicity, and environmental impact necessitate further optimization of AgNP formulations. Advanced delivery strategies, including surface functionalization, biopolymer encapsulation, and stimuli-responsive nanoplatforms, have shown promise in enhancing AgNP stability, selectivity, and controlled release. Additionally, integrating AgNPs with conventional antibiotics or incorporating them into biomedical applications, such as wound dressings and medical coatings, may provide innovative solutions to counteract bacterial resistance while minimizing adverse effects.

Future research should focus on optimizing AgNP synthesis methods, improving their biocompatibility, and conducting rigorous clinical trials to validate their safety and efficacy. Addressing these challenges will be crucial for translating AgNP-based therapies into clinical practice and mitigating the global antibiotic resistance crisis. By harnessing the potential of nanotechnology, AgNPs could play a transformative role in the development of novel antimicrobial strategies, offering a sustainable and effective approach to combat MDR bacterial infections.
